# A prospective cohort study of the accuracy and safety of robot-assisted minimally invasive spinal surgery

**DOI:** 10.1186/s12893-022-01503-4

**Published:** 2022-02-11

**Authors:** Mingxing Fan, Yanming Fang, Qi Zhang, Jingwei Zhao, Bo Liu, Wei Tian

**Affiliations:** 1 Spine Department, Beijing Jishuitan Hospital, Beijing, China; 2 Beijing Key Laboratory of Robotic Orthopaedics, Beijing, China

## Abstract

**Background:**

Robot-assisted open surgery (RA-OS) is now commonly used in traditional open-exposure spinal screw placement surgery. With the help of robots, robot-assisted minimally invasive surgery (RA-MIS) can achieve less bleeding and less tissue damage in percutaneous screw insertion. While the research comparing the safety and accuracy of screw placement between RA-MIS and RA-OS is insufficient. This study aims to compare the effects of RA-MIS and RA-OS in thoracic and lumbar spine.

**Methods:**

This was a prospective cohort study evaluating 208 patients undergoing robot-assisted screw insertions from July 2020 to September 2021. Age, BMI, gender, screws accuracy, screws Gertzbein–Robbins grade, small joint invasion and perioperative outcomes (operation time, blood loss, postoperative hospital stay, comorbidity) were collected. A subgroup analysis was also performed according to disease, namely fracture, spondylolisthesis, and disc herniation. Data were analyzed using Stata/MP 14.0. Wilcoxon’s signed rank test, Kruskal–Wallis test and Fisher’s exact test were used for statistical tests and *p* < 0.05 was considered statistically significant.

**Results:**

A total of 1030 screws were inserted; 368 minimally invasive screws and 662 open screws. The acceptability of screw insertion in the RA-MIS and RA-OS was 97.3% and 95.6% respectively. There was no statistical difference between the RA-MIS group and RA-OS group in age (*p* = 0.106), gender (*p* = 0.074), BMI (*p* = 0.181) and comorbidity (*p* = 0.203). Compared with RA-OS, RA-MIS had less blood loss (*p* < 0.001) and shorter postoperative hospital stay (*p* = 0.008). In the minimally invasive surgery group, the fracture subgroup had smaller screw deviation, less blood loss, and shorter operation time compared with the other subgroups (*p* < 0.01). Specifically, RA-MIS significantly reduced the postoperative hospital stay of patients with spondylolisthesis compared with RA-OS (*p* < 0.01).

**Conclusion:**

RA-OS and RA-MIS had equal accuracy and safety. Compared with open surgery, minimally invasive surgery reduced blood loss in each subgroup and shortened the postoperative hospital stay in the spondylolisthesis subgroup. Compared with the other subgroups under minimally invasive surgery, the fracture subgroup had less blood loss and shorter operation time.

*Clinical trial registration:* NCT04040868. Registered 1 March 2019, https://clinicaltrials.gov/ct2/show/NCT04040868?cond=Accuracy+Study+of+Robot-assisted+Screw+Insertion+in+Spinal+Surgery&draw=2&rank=1.

## Background

Spine surrounds the spinal cord and nerves, playing important roles in support, protection, and movement. Spinal diseases can cause spinal cord and nerve damage that seriously affect patients’ health and quality of life [10, 18]. Pedicle screw internal fixation technology is one of the most important methods to restore the spine to its pre-injury structure and function. In traditional open spinal surgery, some screws have large deviations from the expected path owing to the narrow intraoperative view, complex anatomical structures, and insufficient surgeons’ proficiency. Screw placement accuracy in open surgery is low and ranges from 69 to 94% [6, 16]. Fortunately, the emergence of orthopedic robots has solved this problem. Compared with traditional spine surgery, orthopedic robot-assisted surgery has higher screw placement safety, with an accuracy of between 94.5 and 99% [8, 11, 12], which has also reduced the possibility of nerve damage secondary to inaccurate pedicle screw placement.

Traditional open surgery requires stripping of a large amount of paravertebral muscle and soft tissue, which may lead to several postoperative complications. Additionally, tissue damage and adhesion may cause postoperatively back stiffness and muscle weakness. With the assistance of robots, doctors can perform a minimally invasive surgical method of percutaneous pedicle screw fixation called minimally invasive surgery. It has the advantages of less soft tissue damage, less intraoperative bleeding, shorter hospital stay, and improved quality of life. [8, 14, 20, 21]. However, comparison studies of the effects of RA-MIS vs RA-OS are preliminary, and evaluations of these approaches for different spinal diseases is lacking. There is still no definite answer to whether robot-assisted surgery can be used in different spinal diseases safely and effectively due to different pathophysiological mechanisms.

In this study, we prospectively collected data of 208 patients with thoracic or lumbar spinal disease in our Department of Spine Surgery from July 2020 to September 2021. Patients were divided into RA-MIS and RA-OS groups. Differences in the accuracy, safety, and perioperative outcomes were evaluated according to disease subgroups. This article laid the foundation for studying the clinical effects of RA-MIS and RA-OS in different diseases.

## Methods

### Study design and patient selection

We prospectively enrolled patients in Spine Surgery Department from July 2020 to September 2021 and divided patients into RA-MIS and RA-OS groups according to disease condition and patients’ willing. The sample size was estimated using a non-inferiority test of two independent samples. The test level was set at 0.025, test power was 0.2, and the loss to follow-up ratio was 0. According to a ratio of 1:1.5, 81 patients in the RA-MIS group and 121 patients in the RA-OS were needed. The final total number of enrolled patients was 208 patients, including 79 in the RA-MIS group and 129 in the RA-OS group, with a total of 1030 screws inserted.

The inclusion criteria were as follows: patients aged 18–85 years undergoing robot-assisted pedicle screw internal fixation owing to thoracic or lumbar diseases. The exclusion criteria were as follows: patients with previous spinal surgery; history of tumors or tuberculosis; multiple traumas; concurrent disease, such as severe hypertension and heart disease; and patients not suitable to undergo robotic screw placement. The patients were further divided into fracture, spondylolisthesis, and disc herniation subgroups according to the diagnosis at admission. We defined the fracture subgroup as patients with pain symptoms, malformations, abnormal activities, or bone friction feeling, with imaging examinations showing that the integrity and continuity of the spine was interrupted. We defined the spondylolisthesis subgroup as patients with symptoms related to spondylolisthesis, with imaging studies indicating that a vertebral body had slipped position relative to its adjacent vertebral bodies. We defined the disc herniation subgroup as patients presenting with symptoms related to a herniated vertebral disc, with imaging examinations suggesting that the disc was herniated. This study was approved by the Ethics Committee and patients’ agreement.

### Robotic system and surgical procedure

**Fig. 1:**
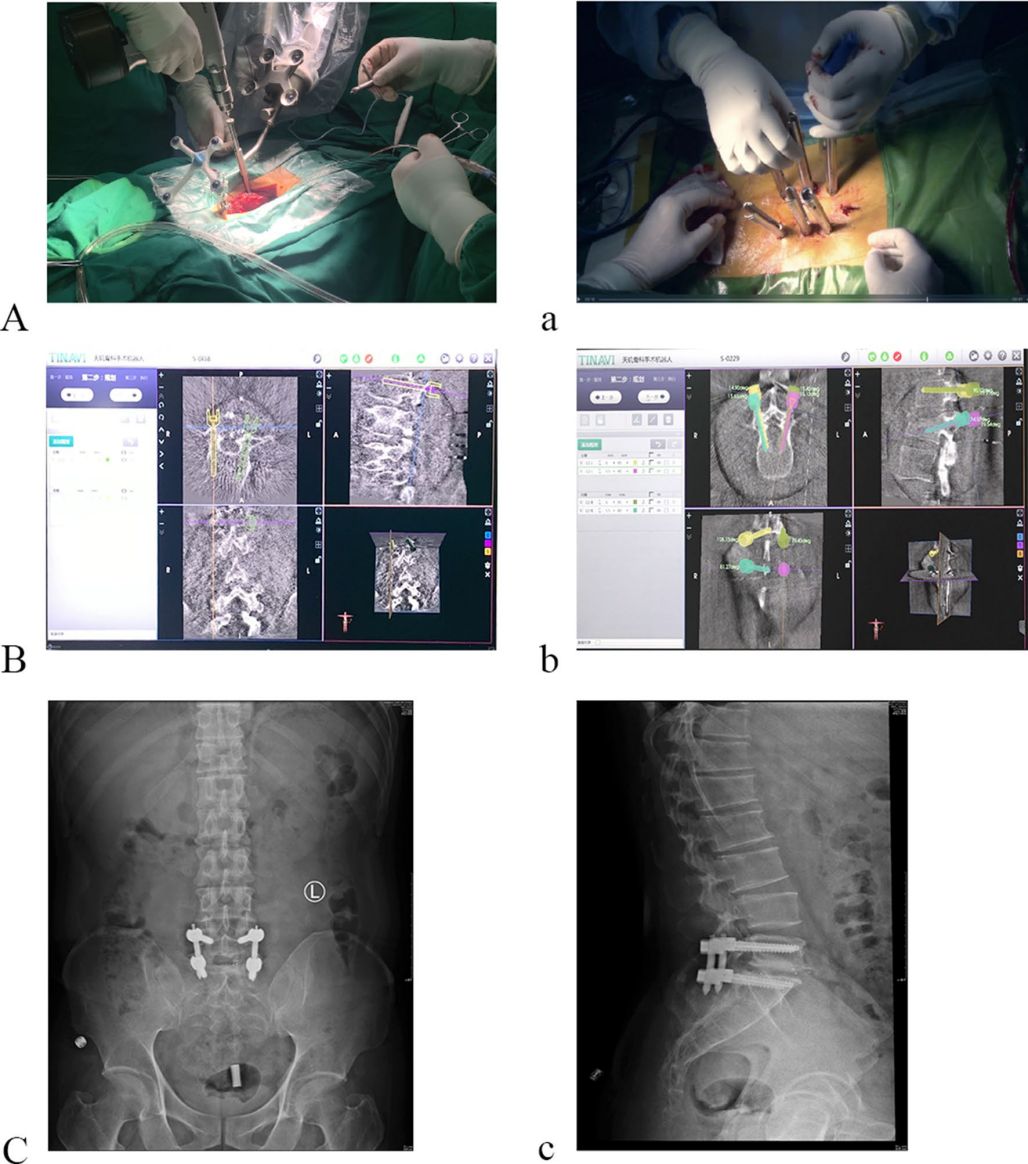
Clinical photographs, intraprocedural imaging and radiographs. **A** Surgery photo of RA-OS; **a** surgery photo of RA-MIS; **B**: Screw insertion design of cervical spine; **b** Screw insertion design of lumbar spine; **C** and **c** radiographs after lumbar surgery

Robot-assisted surgery was performed using the TiRobot orthopedic robot system (TINAVI Medical Technologies, Beijing, China). Orthopedic surgery robot system is composed of host, mechanical arm, surgery planning and control software, optical tracking system, main control trolley and navigation and positioning toolkit. Its operation involves image and optical data acquisition, image registration, surgical planning, mechanical positioning and other steps. The robot uses intraoperative images for surgical planning, and uses robotic arm for movement and holding to achieve precise positioning of implants. Its working process mainly includes 4 steps: (1) Surgical planning, the doctor uses the supporting surgical planning and control software to design the surgical path on the intraoperative images, and select the appropriate implant; (2) Spatial calibration, obtain the spatial coordinates of the surgical path through a certain positioning algorithm and device; (3) Path positioning, control the robot to automatically move according to the spatial coordinates of the surgical path, and move the surgical tool to the target surgical path; (4) Auxiliary surgery, the doctor performs surgical operations under the guidance of a robotic arm (Fig. 1).

**Fig. 2:**
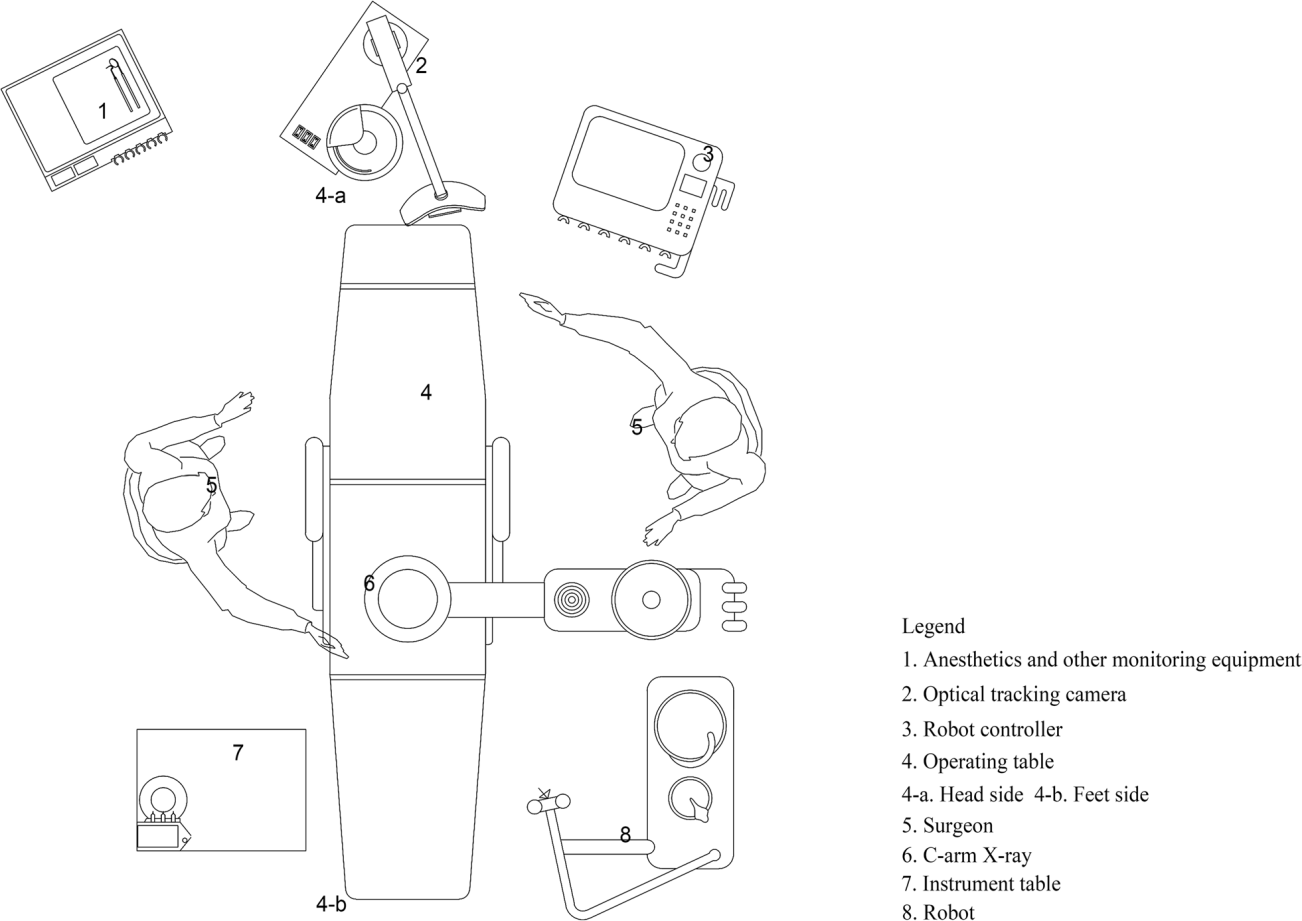
Schematic diagram of the placement of the patient and the robotic equipment

**Fig. 3:**
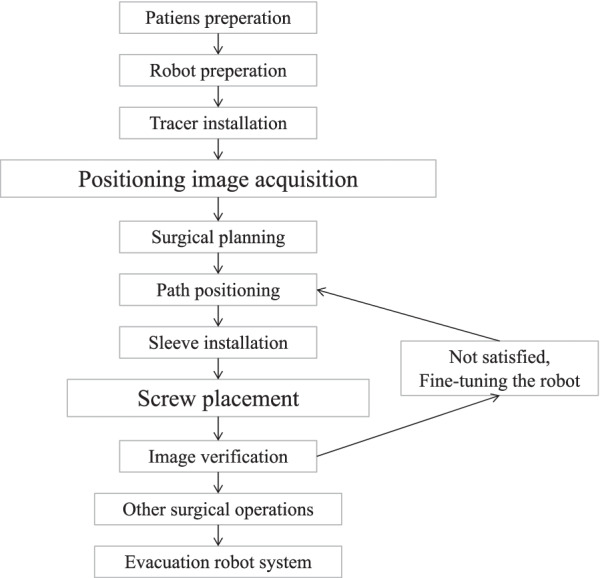
Operative steps in robotic surgery

The patient was placed in the prone position on the X-ray/operating table (Fig. 2). After inducing general anesthesia, a C-arm X-ray machine (ARCADIS Orbic 3D C-arm; Siemens, Erlangen, Germany) was used to locate the operating area. RA-OS exposed the surgical field according to the requirements of traditional surgery. (RA-MIS firstly exposed and fixed the patient tracer, then exposed the surgical field under robot guidance.) The tracer was usually fixed on the spinous process of the upper vertebral body adjacent to the surgical segment. After image acquisition using intraoperative real-time three-dimensional navigation, surgeons planned and designed the surgery using the robot control software and selected the spine level, screw diameter, length, direction and angle. After the guide was installed, the robot-arm advanced to the planned position, and a sleeve was placed on the guide to monitor the positioning accuracy in real time. A powered system was used to drill into the K-wire along the sleeve. Hollow screws can be inserted directly along the K-wire; with ordinary screws, a hollow drill was used to prepare the screw channel before insertion. After screw insertion, C-arm X-ray fluoroscopy was used to assess screw placement, and bilateral connecting rods were installed followed by suturing the incision (Fig. 3).

### Outcome measures

This study analyzed the patients’ basic demographic characteristics, namely sex, age, body mass index (BMI), and perioperative outcomes, namely operative time, intraoperative blood loss, postoperative hospital stay, and comorbidity. According to postoperative computed tomography (CT) findings, the number of screw facet joint invasions, screw deviation (distance that the screw deviated from the designed trajectory), and screw safety were assessed. Gertzbein and Robbins scale [7] was used for estimating screw safety as follows: Grade A: the screw is completely in the pedicle; Grade B: the distance of the screw breaking through the pedicle cortex is < 2 mm; Grade C: the distance of the screw breaking through the pedicle cortex is ≥ 2 mm and < 4 mm; Grade D: the distance of the screw breaking through the pedicle cortex is ≥ 4 mm and < 6 mm; and Grade E: the distance of the screw breaking through the pedicle cortex is > 6 mm. We calculated the acceptability rate of screw placement using the following formula: [(number of grade A screws + number of grade B screws) / total number of screws × 100%].

### Statistical analysis

Data were analyzed using Stata/MP 14.0 (College Station, TX, USA). The Shapiro–Wilk’s test was used for normally-distributed data, with analysis of variance (ANOVA). Measurement data conforming to normal distribution and homogeneity of variance was expressed as mean ± standard deviation. Measurement data that not conforming to normal distribution or homogeneity of variance was expressed as median (75% quartile–25% quartile). Wilcoxon’s signed rank test was performed for comparisons between two groups, and the Kruskal–Wallis test was performed for comparisons between multiple subgroups. Numerical data are expressed as frequency (percent). Comparisons between groups were also performed using Fisher’s exact test or the chi-square test, and the rank mean difference (RMD) was calculated. *p* < 0.05 was considered statistically significant.

## Results

### Demographic data

Two hundred and eight patients were enrolled in this study with 79 in the RA-MIS group and 129 in the RA-OS group. A total of 1030 screws were inserted. There was no statistical difference between the RA-MIS group and RA-OS group for age [56 years (64–50) vs 60 years (64–54), *p* = 0.106], gender, BMI [25.69 kg/m^2^ (27.68–23.53) vs 25 kg/m^2^ (27.63–22.58), *p* = 0.181] and comorbidity [1 vs 0, *p* = 0. 0.203)] (Table 1).

**Table 1:** Demographic data, perioperative outcomes and screw placement results for the RA-MIS and RA-OS

	Minimally invasive surgery (N = 79)	Open surgery (N = 129)	Statistics	*p* Value
Age (year)	56 (64–50)	60 (64–54)	Z = − 1.615	0.106
Gender			χ^2^ = 3.2020	0.074
Male	40 (44.6%)	49 (44.4%)		
Female	39 (55.4%)	80 (55.6%)		
BMI (kg/m^2^)	25.69 (27.68–23.53)	25 (27.63–22.58)	Z = 1.1139	0.181
Operation time (minutes)	150 (180–100)	135 (180–120)	Z = − 0.034	0.973
Intraoperative blood loss (mL)	100 (200–50)	200 (400–200)	Z = − 6.347	< 0.001
Postoperative hospital stay (days)	5 (6–4)	5 (7–4)	Z = − 2.658	0.008
Comorbidity	1	0	χ^2^ = 1.6203	0.203
Number of screws	368	662	χ^2^ = 0.0000	1.000
Thoracic spine	50(13.6%)	90 (13.6%)		
Lumbosacral Spine	318 (86.4%)	572 (86.4%)		
Small joint invasion	9 (2.4%)	20 (3.0%)	Fisher	0.697
GRS grade			Fisher	0.470
Grade A	331 (90.0%)	583 (88.1%)		
Grade B	27 (7.3%)	50 (7.5%)		
Grade (A + B)	358 (97.3%)	633 (95.6%)		
Grade C	9 (2.4%)	25 (3.8%)		
Grade D	1 (0.3%)	4 (0.6%)		
Screw deviation (mm)	1.37 (2.05–0.86)	1.34 (2.21–0.90)	Z = − 1.048	0.2948

Age, BMI, operation time, blood loss, postoperative hospital stay and screw deviation were expressed as median (75% quartile–25% quartile). Gender, comorbidity, number of screws, facet joint invasion and Gertzbein and Robbins (GRS) grade are expressed as frequency (percentage). *BMI* body mass index

### Perioperative outcomes and Screw characteristics in generally

There was no statistical difference in operation time between the two groups. Blood loss in the RA-MIS group was less than that in the RA-OS group [100 mL (200–50) vs 200 mL (400–200), *p* < 0.001] and the postoperative hospital stay [5 days (6–4) vs 5 days (7–4), *p* = 0.008] was shorter (Table 1). One patient developed postoperative superficial incision infection in the RA-MIS group, and was cured through local wound disinfection and short-term antibiotic treatment. There was no statistical difference between the two groups on comorbidities (*p* = 0.203). No others critical issues were identified else.

A total of 1030 screws were inserted: 368 screws in the RA-MIS group, namely 50 in the thoracic spine and 318 in the lumbosacral spine. Among these, there were 331 grade A screws (90.0%) and 27 grade B screws (7.3%). In the RA-OS group, 662 pedicle screws were inserted with 90 in the thoracic spine and 572 in the lumbosacral spine. Among these screws, 583 (88.1%) were grade A screws, and 50 (7.5%) were grade B screws. The acceptability rate of screw placement was 97.3% in the RA-MIS and 95.6% in the RA-OS (Table 1). There was no statistical difference between the two groups regarding screw distribution according to the spinal levels (χ^2^ = 0.000; *p* = 1.000), number of small joint invasions (Fisher’s, *p* = 0.697), safety classification (Fisher’s, *p* = 0.470) and screw deviation (Z =  − 1.048; *p* = 0.295).

### Screw characteristics in the subgroups

**Fig. 4:**
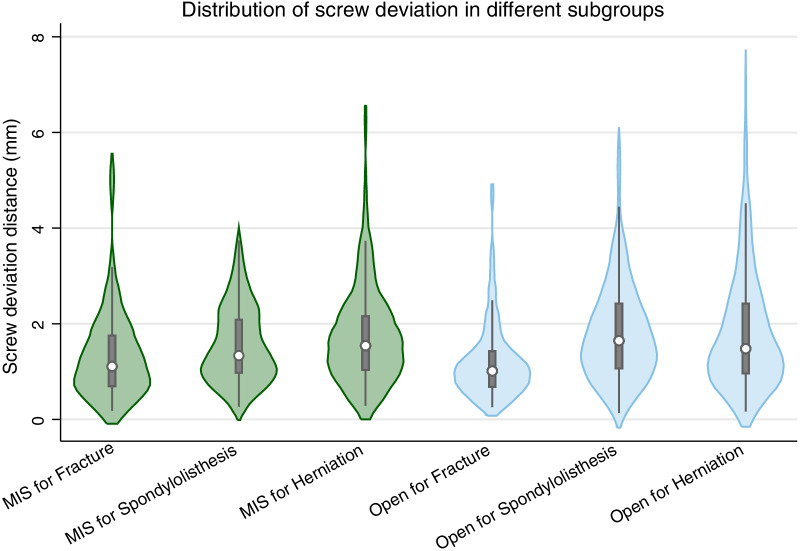
Violin plots of the screw deviation distance distributions. The ordinate is the deviation distance, the width of the violin is the relative frequency distribution, and the center of the violin is the box plot. *MIS* minimally invasive surgery

Screw deviation was statistically different in the subgroup of RA-MISs (Table 2). Screw deviation in fracture subgroup was smaller than that in spondylolisthesis subgroup [1.11 mm (1.75–0.69) vs 1.33 mm (2.09–0.97), RMD = 37.73, *p* < 0.003] and disc herniation subgroup [1.11 mm (1.75–0.69) vs 1.54 mm (2.15–1.03), RMD = 53.04, *p* < 0.001]. A similar trend was observed in the RA-OS group. Screw accuracy in fracture patients was significantly higher than in the spondylolisthesis patients [1.01 mm (1.42–0.68) vs 1.64 mm (2.42–1.06), RMD = 132.10, *p* < 0.001] and the disc herniation patients [1.01 mm (1.42–0.68) vs 1.47 mm (2.42–0.96), RMD = 113.74, *p* < 0.001]. Additionally, the precision of RA-MIS was higher than for RA-OS [1.33 mm (2.09–0.97) vs 1.64 mm (2.42–1. 06); *p* = 0.036] in the spondylolisthesis subgroup (Fig. 4). In the fracture subgroup or disc herniation subgroup, the choice of MIS or RA-OS had no significant statistically impact on screw deviation.

**Table 2:** Screw placement in the disease subgroups

		Fracture	Spondylolisthesis	Disc herniation	Statistics	*p* Value
Number of patients	Minimally Invasive	24	28	27	χ^2^ = 4.5278	0.104
	Open	24	46	59		
Number of screws	Minimally Invasive	122	120	126	χ^2^ = 10.9273	0.006
	Open	160	222	280		
Grade (A + B)/ Total	Minimally Invasive	121/122 (99.18%)	115/120 (95.8%)	122/126 (96.83%)		
	Open	157/160 (98.13%)	213/222 (95.95%)	269/280 (96.07%)		
Screw deviation (mm)	Minimally Invasive	1.11 (1.75–0.69) *∆	1.33 (2.09–0.97)	1.54 (2.15–1.03)	χ^2^ = 16.240	< 0.001
	Open	1.01 (1.42–0.68) *∆	1.64 (2.42–1.06)	1.47 (2.42–0.96)	χ^2^ = 50.400	< 0.001

Screw deviation was expressed as median (75% quartile–25% quartile), and the number of screws and patients were expressed in frequency. *RMD* rank means difference

*Represents statistical significance compared with the spondylolisthesis group (*p* < 0.05), and ∆ represents statistical significance compared with disc herniation group (*p* < 0.05) undergoing same surgical method (minimally invasive group or open group)

### Perioperative outcomes in the subgroups

There were statistical differences in intraoperative blood loss and operation time between the subgroups undergoing RA-MIS. Intraoperative blood loss was lower in the fracture subgroup than in the spondylolisthesis subgroup [50 mL (90–50) vs 100 mL (200–50), RMD = 16.57; *p* = 0.004)] and disc herniation subgroup [50 mL (90–50) vs 200 mL (200–100), RMD = 25.55; *p* < 0.001]. Similarly, operation time in the fracture subgroup was shorter than in the spondylolisthesis subgroup [100 min (135–90) vs 150 min (180–150), RMD = 20.22; *p* < 0.001] and disc herniation subgroup [100 min (135–90) vs 150 min (225–120), RMD = 17.30; *p* = 0.003]. There were also statistical differences in intraoperative blood loss and postoperative hospital stay in the different subgroups in the RA-OS group. Intraoperative blood loss in the fracture subgroup was less than in the spondylolisthesis subgroup [100 mL (200–50) vs 200 mL (400–200), RMD = 37.41; *p* < 0.001] and the disc herniation subgroup [100 mL (200–50) vs 200 mL (400–200), RMD = 36.44; *p* < 0.001]. Postoperative hospital stay in the spondylolisthesis subgroup was longer than that in the fracture subgroup [120 min (170–112.5) vs 135 min (180–120), RMD = 29.00, *p* = 0.001] and disc herniation subgroup [120 min (170–112.5) vs 150 min (180–120), RMD = 20.10; *p* = 0.003]. Compared with RA-OS, RA-MIS was associated with less intraoperative blood loss in each subgroup (*p* < 0.001) and shorter postoperative hospital stay in the spondylolisthesis subgroup (Z =  − 3.491; *p* < 0.001) (Table 3).

**Table 3:** Perioperative outcomes in the disease subgroups

		Fracture	Spondylolisthesis	Disc herniation	Statistics	*p* Value
Intraoperative blood loss (mL)	Minimally Invasive	50 (90–50) *∆	100 (200–50)	200 (200–100)	χ^2^ = 17.665	< 0.001
	Open	100 (200–50) *∆	200 (400–200)	200 (400–200)	χ^2^ = 21.381	< 0.001
Operation time (minutes)	Minimally Invasive	100 (135–90) *∆	150 (180–150)	150 (225–120)	χ^2^ = 12.831	0.002
	Open	120 (170–112.5)	135 (180–120)	150 (180–120)	χ^2^ = 1.413	0.493
Postoperative hospital stay (days)	Minimally Invasive	4 (5–4)	5 (5.5–4)	5 (6–4)	χ^2^ = 5.360	0.069
	Open	4.5 (6.5–3.5) *	6 (7–5)	5 (6–4) *	χ^2^ = 12.248	0.002

Operation time, blood loss, and postoperative hospital stay are expressed as median (75% quartile–25% quartile). *RMD* rank means difference

*Represents statistical significance compared with the spondylolisthesis group (*p* < 0.05), and ∆ represents statistical significance compared with disc herniation group (*p* < 0.05) undergoing same surgical method (minimally invasive group or open group)

## Discussion

Internal screw fixation is an important method to treat spinal injuries and instability. With freehand screw insertion, screw is easier to deviate from the designed trajectory owing to the narrow visual field, and may enter the spinal canal and damage the spinal cord or the nerve root. Most foot drop cases after lumbar internal fixation are related to this issue [4, 9]. The incidence of nerve root irritation during pedicle screw insertion can be as high as 21%, of which a considerable number of patients have poorly positioned screws but are asymptomatic [2]. In addition, screws of inappropriate length may penetrate the vertebra, injuring blood vessels or adjacent organs. With the development of robot-assisted screw placement technology, the length and angle of screw placement are finely controlled. The accuracy and safety of screw placement have been further improved, and iatrogenic injuries have gradually decreased. Compared with traditional freehand screw insertion, the acceptability of robot-assisted screw insertion can reach 95–99% [11, 12], which matches the 97.3% acceptability rate in our RA-MIS group and 95.6% in the RA-OS group.

Soft tissues connect bony structures, ligaments, discs, and muscles, and are important structures in maintaining spinal stability. Damage to the spinal accessory tissues reduces surgical efficacy and increases the risk of complications. The incidence of surgical infections after instrument surgery is relatively high, which may be related to the greater soft tissue dissection, large surgical wounds, and use of instruments [17, 22]. Minimally invasive spine surgery decreases intraoperative blood loss, shortens postoperative hospital stay, and reduces tissue damage. Robot-assisted minimally invasive spine surgery is a new development direction in spinal surgery [14, 20].

In this study, there was no statistical difference in screw deviation, Gertzbein and Robbins grade distribution, and facet joint invasion between the RA-MIS and RA-OS groups. Screw deviation in the RA-MIS group was smaller than that in the RA-OS group but with no statistical difference, which was better than the 2.0 ± 1.2 deviation reported by van Dijk et al. using the Mazor robot [19]. Regarding small joint invasion, the 2–3% invasion rate in our study was better than the 7% invasion rate in the robot group reported by Archavlis et al. [1]. This showed that the positioning accuracy and safety of the TiRobot has reached the international robotic level. While preserving more tissues around the spine, RA-MIS can maintain the same accuracy and safety as RA-OS.

Regarding the perioperative outcomes in this study, the RA-MIS group had statistically significantly less intraoperative blood loss and shorter postoperative hospital stay than the RA-OS group. Less blood loss may be related to the smaller surgical incision, less muscle dissection, and less intraoperative vascular damage, intraoperatively. The shorter postoperative hospital stay may have resulted from less postoperative bleeding and drainage [13, 15]. Compared with RA-OS, RA-MIS had statistically significant effects in reducing intraoperative blood loss. Additionally, one patient in the RA-MIS group underwent postoperative expansion surgery compared with none in the RA-OS group. This may be related to wound infection caused by the patient's second-degree obesity (BMI = 31.25) [5].

We also performed a subgroup analysis by disease and compared the RA-MIS and RA-OS groups. For RA-MIS, screw deviation in the fracture subgroup was smaller than that in the spondylolisthesis subgroup and the disc herniation subgroup. The 99.18% screw placement acceptance rate in the fracture subgroup was better than the 97.7% acceptance rate described by Lin et al. [13], and the rate in our spondylolisthesis subgroup was better than data reported by Cui et al. (95.8% vs 93.8%, respectively) [3, 13]. The difference between the subgroups may be related to the degree of damage to the vertebral body. In particular, for patients with spondylolisthesis, the screw precision in the RA-MIS group was higher (*p* = 0.036), and the postoperative hospital stay was shorter (*p* < 0.001) compared with RA-OS. This may be related to the better preservation of intervertebral joint structures and less muscle damage in patients undergoing MIS. Regarding the perioperative outcomes, intraoperative blood loss was lower and operation time was shorter in the fracture subgroup than in the other groups. This may be because of easier fracture treatment and the relatively short postoperative immobilization time.

In summary, our results indicated that RA-MIS and RA-OS have equal accuracy and safety. MIS was associated with less intraoperative blood loss in each subgroup and a shorter postoperative hospital stay in the spondylolisthesis subgroup. Our subgroup analysis of MIS showed that the fracture group experienced less blood loss, shorter operation time, and shorter postoperative hospital stay compared with the spondylolisthesis group and disc herniation group. This study has the following shortcomings: there were few relevant published references; therefore, comparisons with others studies were not comprehensive. Additionally, we did not evaluate bone density, which may have caused confounding bias.

## Conclusions

RA-OS and RA-MIS had the same accuracy and safety. Compared with open surgery, minimally invasive surgery reduced blood loss in all fracture subgroup, spondylolisthesis subgroup as well as disc herniation subgroup, and shortened the postoperative hospital stay in the spondylolisthesis subgroup. Compared with the other subgroups under minimally invasive surgery, the fracture subgroup had less blood loss and shorter operation time. This paper studied the effect of robotic-assisted minimally invasive surgery screws in different diseases, and provided a basis for economic benefit research.

## Data Availability

The datasets used and/or analysed during the current study are available from the corresponding author on reasonable request.

## Abbreviations

RA-MIS: Robot-assisted minimally invasive surgery
RA-OS: Robot-assisted open surgery
BMI: Body mass index
CT: Computed tomography
ANOVA: Analysis of variance
RMD: Rank mean difference
